# PGC‐1α affects aging‐related changes in muscle and motor function by modulating specific exercise‐mediated changes in old mice

**DOI:** 10.1111/acel.12697

**Published:** 2017-10-25

**Authors:** Jonathan F. Gill, Gesa Santos, Svenia Schnyder, Christoph Handschin

**Affiliations:** ^1^ Biozentrum University of Basel Basel Switzerland

**Keywords:** aging, exercise, mitochondria, motor function, PGC‐1α, sarcopenia, skeletal muscle

## Abstract

The age‐related impairment in muscle function results in a drastic decline in motor coordination and mobility in elderly individuals. Regular physical activity is the only efficient intervention to prevent and treat this age‐associated degeneration. However, the mechanisms that underlie the therapeutic effect of exercise in this context remain unclear. We assessed whether endurance exercise training in old age is sufficient to affect muscle and motor function. Moreover, as muscle peroxisome proliferator‐activated receptor γ coactivator 1α (PGC‐1α) is a key regulatory hub in endurance exercise adaptation with decreased expression in old muscle, we studied the involvement of PGC‐1α in the therapeutic effect of exercise in aging. Intriguingly, PGC‐1α muscle‐specific knockout and overexpression, respectively, precipitated and alleviated specific aspects of aging‐related deterioration of muscle function in old mice, while other muscle dysfunctions remained unchanged upon PGC‐1α modulation. Surprisingly, we discovered that muscle PGC‐1α was not only involved in improving muscle endurance and mitochondrial remodeling, but also phenocopied endurance exercise training in advanced age by contributing to maintaining balance and motor coordination in old animals. Our data therefore suggest that the benefits of exercise, even when performed at old age, extend beyond skeletal muscle and are at least in part mediated by PGC‐1α.

**Abbreviations**
ATP6: ATP synthase 6
COX1: cytochrome C oxidase I
ND1: NADH dehydrogenase subunit 1
OXPHOS: oxidative phosphorylation
PGC‐1α: peroxisome proliferator‐activated receptor γ coactivator 1α
TA: tibialis anterior
TBP: TATA binding protein

## 1 INTRODUCTION

Aging is a progressive and inevitable biological process leading to a decay and dysfunction of organs. Skeletal muscle tissue is prominently affected by this decline, as characterized by a loss of strength and mass starting in the 4th decade in humans, a process known as sarcopenia (Nair, 2005). Age‐related loss of muscle function is further exacerbated by reduced balance and motor coordination resulting in avoidance of physical ability, increased frailty, elevated number of falls, and thus bone fractures (Lord, Clark, & Webster, 1991), highly predictive for hospitalization and morbidity (Rolland et al., 2008). Decreased mobility is associated with an impaired ability to perform daily tasks or engage in social interactions and thus markedly affects quality of life (Landi et al., 2017). In addition, muscle dysfunction is associated with many age‐associated metabolic disorders such as insulin resistance, type 2 diabetes, or hypertension, thereby increasing the risk of cardiovascular death (Nair, 2005). While multiple events are involved in muscle aging (Marcell, 2003), mitochondrial dysfunction has been strongly linked to sarcopenia (Short et al., 2005).

Exercise is the most efficient intervention to improve muscle function and whole‐body metabolism (Egan & Zierath, 2013), while physical inactivity increases the risk for many chronic diseases associated with aging (Booth, Roberts, & Laye, 2012). Endurance exercise training accordingly helps to ameliorate age‐related skeletal muscle dysfunctions (Cartee, Hepple, Bamman, & Zierath, 2016), not only limited to the preservation of muscle mass and strength, but also balance, motor coordination and mobility (Garatachea et al., 2015). Furthermore, exercise also reduces the age‐related decline in insulin sensitivity (Cartee et al., 2016). Finally, physical activity maintains normal mitochondrial function in older adults (Gouspillou et al., 2014). Mitochondrial activity and other exercise‐induced phenotypic changes of skeletal muscle are controlled by the peroxisome proliferator‐activated receptor γ coactivator 1α (PGC‐1α). For example, PGC‐1α is a master regulator of mitochondrial biogenesis (Schreiber et al., 2004; Wu et al., 1999), mitochondrial function (Wu et al., 1999), mitochondrial dynamics (Cannavino et al., 2015), fatty acid oxidation (Hoeks et al., 2012), and anti‐oxidative processes (St‐Pierre et al., 2006). Interestingly, PGC‐1α levels are increased by exercise in muscle (Safdar et al., 2011) and PGC‐1α is considered to be a key player in mitochondrial changes induced by exercise (Safdar et al., 2011; Ventura‐Clapier, Mettauer, & Bigard, 2007). In addition, PGC‐1α extends health span and life span of a mouse model of premature aging arising from mitochondria defects (Sahin et al., 2011). Importantly, in line with the mitochondrial decline, PGC‐1α expression decreases during muscle aging in different species, including humans (Ghosh et al., 2011; Kang, Chung, Diffee, & Ji, 2013; Short et al., 2005; Vina et al., 2009). Moreover, PGC‐1α improves various muscle disorders, for example, denervation‐induced fiber atrophy (Sandri et al., 2006) or Duchenne muscular dystrophy (Handschin, Kobayashi et al., 2007). Of note, conclusions about the function of PGC‐1α in the context of natural aging are difficult to extrapolate from premature aging models, for example, those with severe mitochondrial impairment. Moreover, other studies reported unchanged muscle PGC‐1α levels despite muscle atrophy in active humans (Gouspillou et al., 2014). Therefore, the role of PGC‐1α in muscle aging remains an open question and needs to be addressed.

In this work, we investigated the impact of endurance exercise training at old age on muscle function, age‐related motor dysfunction, and whole‐body metabolism in 2‐year‐old mice. Moreover, we assessed how muscle‐specific gain of function and loss of function of PGC‐1α affects the aging process in sedentary and trained animals. Previous studies (Kim, Kim, Oh, Kim, & Song, 2013; LeBrasseur et al., 2009) and current investigations performed in our laboratory with young animals showed that 3 months of treadmill endurance training was sufficient to induce significant adaptations in mice. The age of 21 months as starting point was chosen based on the presarcopenic state of mice at that age, and to avoid a potential reduction in lifespan of the PGC‐1α knockout mice. While a number of effects of muscle‐specific gene ablation of PGC‐1α on muscle metabolism in aging have been demonstrated (Sczelecki et al., 2014), we now observed that reduced and elevated PGC‐1α levels accelerate and delay, respectively, broader aspects of muscle aging. Interestingly however, we also show that PGC‐1α modulation is not sufficient to fully control muscle aging as some age‐related muscle dysfunctions are not altered by deletion or overexpression of this coactivator. Moreover, we found that PGC‐1α slightly improves whole‐body metabolism and that many beneficial effects of exercise on mitochondria and muscle function are potentiated by and dependent on this transcriptional coactivator. Finally, we discovered that the effects of exercise and muscle PGC‐1α extend beyond skeletal muscle tissue, for example, by impacting on motor coordination and balance.

## 2 RESULTS

### 2.1 Exercise‐associated mitochondrial improvement in old muscle is dependent on muscle PGC‐1α

**Figure 1:**
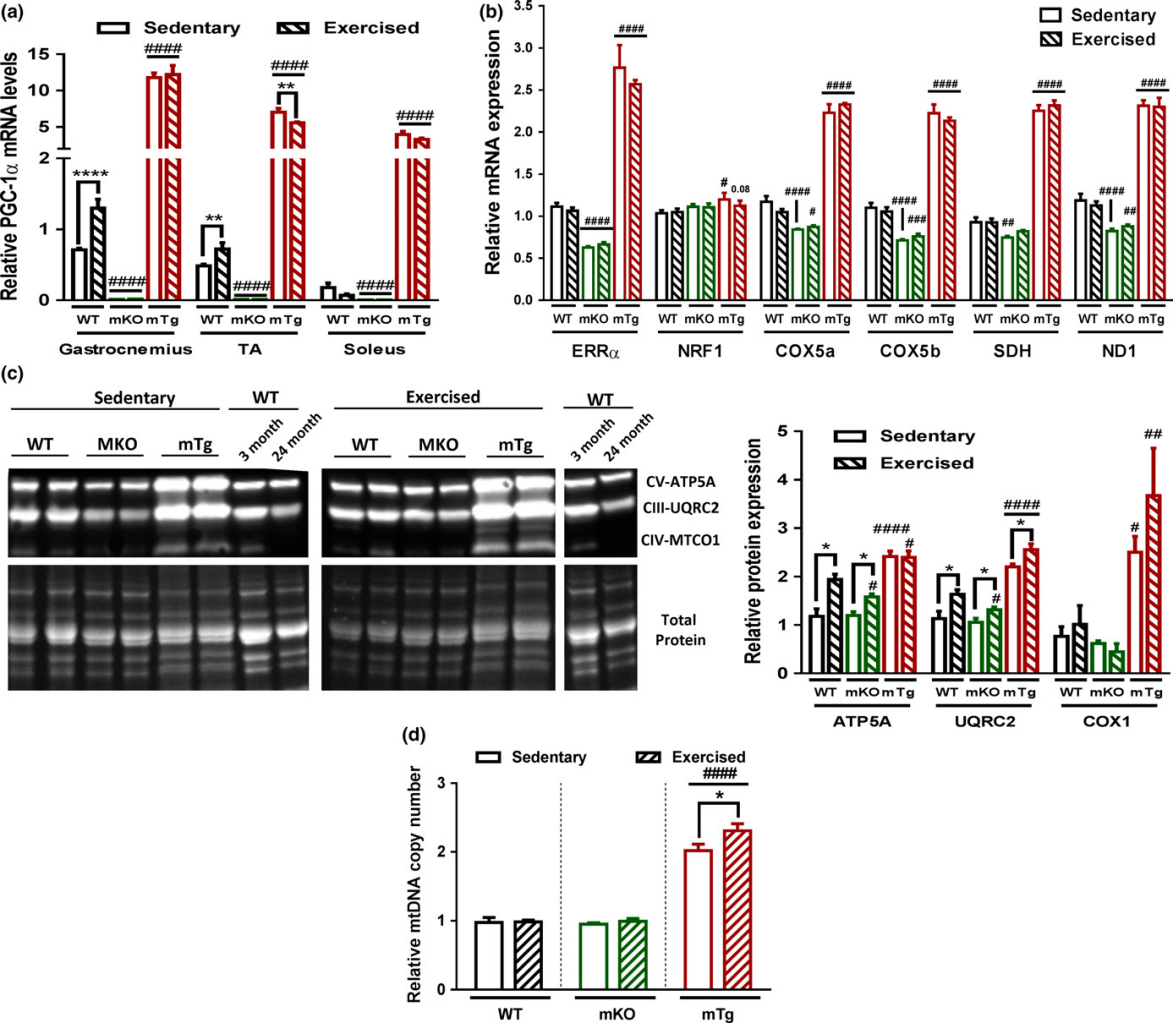
PGC‐1α levels determine exercise‐related mitochondrial improvements (a) Relative PGC‐1α gene expression in different muscles (*n* = 3–6). (b) Relative gastrocnemius mRNA levels of mitochondrial genes (*n* = 6). (c) Quadriceps mitochondrial OXPHOS protein levels (*n* = 6). (d) Mitochondrial mass quantified in gastrocnemius muscle (*n* = 6). Values are mean ± *SEM*. *p < .05; **p < .01; ****p < .0001 indicate statistically significant differences between sedentary and exercised animals of the same genotype, ^#^ *p* < .01; ^##^ *p* < .01; ^###^ *p* < .01; ^####^ *p* < .001 indicate statistically significant differences between genotypes for sedentary and exercised animals

Mitochondrial number and function are boosted by endurance training, while inversely, reduced mitochondrial activity has been associated with muscle aging. We therefore investigated the impact of exercise and modulation of muscle PGC‐1α expression in old skeletal muscle in vivo. Even when only performed at advanced age, endurance exercise training induced PGC‐1α expression in the gastrocnemius and tibialis anterior (TA) muscle of wild‐type (WT) animals (Figure 1a). Surprisingly however, the genotype effect of overexpression and knockout of PGC‐1α on mitochondrial gene expression was not replicated by exercise (Figure 1b). Nevertheless, oxidative phosphorylation (OXPHOS) protein levels were elevated by PGC‐1α and exercise (Figure 1c). Importantly, the exercise‐controlled increase in OXPHOS proteins was blunted in the muscle‐specific PGC‐1α knockout (mKO) animals. Even though mitochondrial gene expression and protein levels were already substantially boosted in muscle‐specific PGC‐1α transgenic (mTg) mice beyond the levels that were achieved by endurance exercise in WT animals, an additional training effect was nevertheless observed in the mTg model in regard to mitochondrial DNA copy numbers (Figure 1d).

### 2.2 Muscle PGC‐1α affects endurance capacity of untrained and trained skeletal muscle in old mice

**Figure 2:**
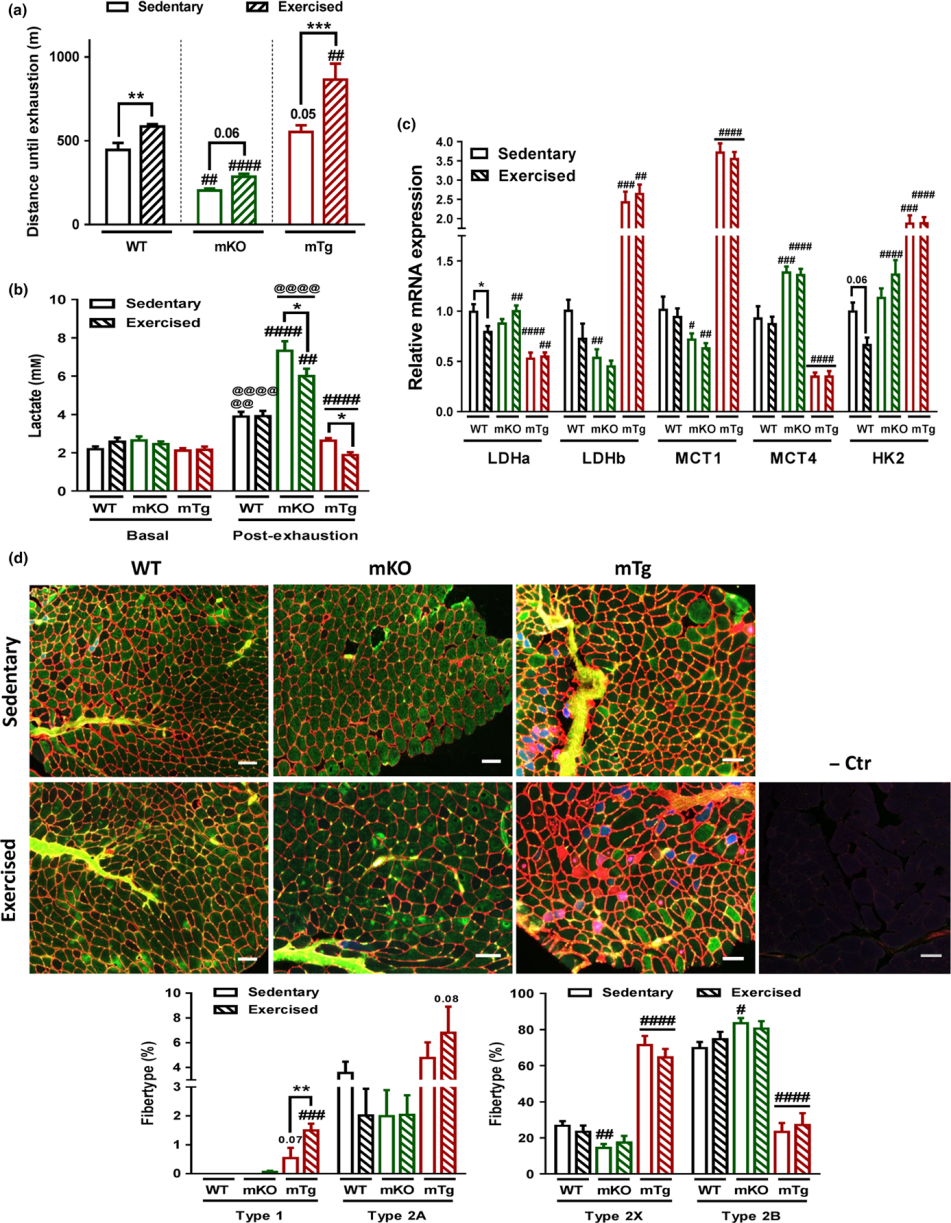
PGC‐1α potentiates exercise‐dependent endurance increase (a) Endurance level of mice challenged on a treadmill (*n* = 8–10). (b) Pre and postexercise blood lactate levels (*n* = 8–10). (c) Relative quadriceps mRNA levels of genes involved in lactate regulation and glycolysis (*n* = 4–6). (d) Representative pictures and quantification of fiber type staining of tibialis anterior muscles (red = type 1 fibers and cell membranes; blue = type 2A fibers; green = type 2B fibers; black = type 2X fibers). Scale bars represent 100 μm (*n* = 4–6). Values are mean ± *SEM*. *p < .05; **p < .01; ***p < .001; indicate statistically significant differences between sedentary and exercised animals of the same genotype, ^#^ *p* < .01; ^##^ *p* < .01; ^###^ *p* < .01; ^####^ *p* < .001 indicate statistically significant differences between genotypes for sedentary and exercised animals, ^@@^ *p* < 0.01; ^@@@@^ *p* < 0.0001 indicate statistically significant differences pre‐ and post‐exhaustion

Mitochondrial function has been linked to endurance performance in skeletal muscle. Based on the observed modulation of mitochondrial properties by exercise and muscle PGC‐1α, we next studied the impact of the corresponding interventions on muscle endurance during aging. In all three genotypes, endurance exercise training improved endurance performance of old mice, even though at lower and higher levels in mKO and mTg animals, respectively (Figure 2a). Similarly, circulating lactate was affected in a genotype‐dependent manner (Figure 2b). Surprisingly, an additional lactate‐lowering effect of endurance exercise training was only observed in mKO and mTg, but not WT mice. In line with the literature, gene expression regulating lactate metabolism and glycolysis was affected in both genotypes, even though these data fail to fully explain the exercise effect on circulating lactate in the mKOs (Figure 2c and Fig. S1). At least in the mTg mice with a reported higher number of oxidative muscle fibers (Handschin, Choi et al., 2007; Lin et al., 2002), this reduction could be based on the exercise‐linked potentiation of the proportion of type IIa and type I muscle fibers, which was not recorded in WT or mKO animals (Figure 2d).

### 2.3 Aging, exercise and muscle PGC‐1α modulate muscle mass and metabolic parameters

**Figure 3:**
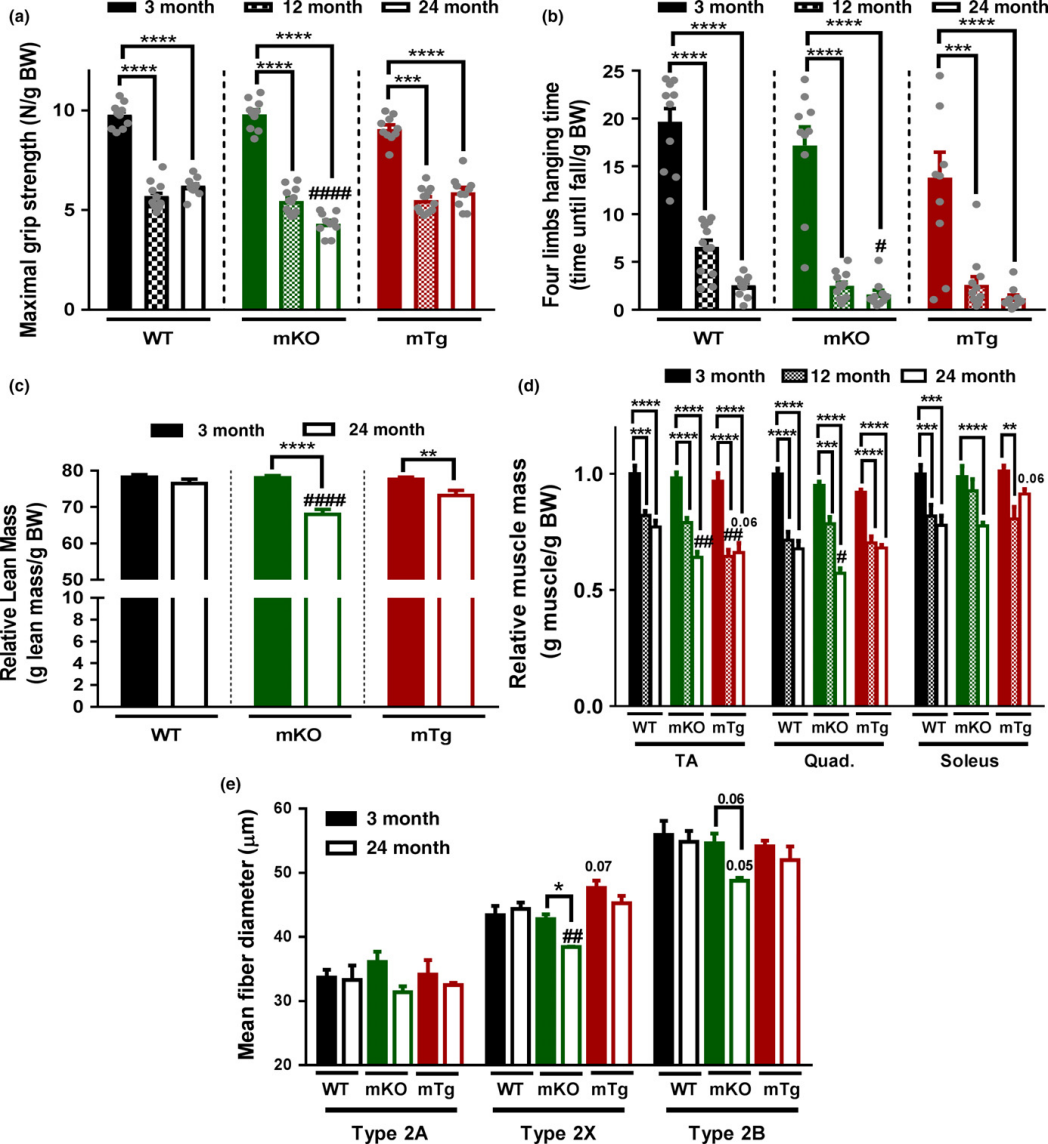
Absence of PGC‐1α accelerate sarcopenia (a) Maximal grip strength (*n* = 10–12). (b) Four limbs hanging time (*n* = 10–12). (c) Lean relative to body mass (*n* = 10). (d) Muscle mass relative to body weight (*n* = 5–6). (e) Tibialis anterior fiber type specific minimum ferret diameter (*n* = 3–4). Values are mean ± *SEM*. *p < .05; **p < .01; ***p < .001; ****p < .0001 indicate statistically significant differences between young and old animals of the same genotype. ^#^ *p* < .01; ^##^ *p* < .01; ^####^ *p* < 0.001 indicate statistically significant differences between genotypes of age‐matched animals

**Figure 4:**
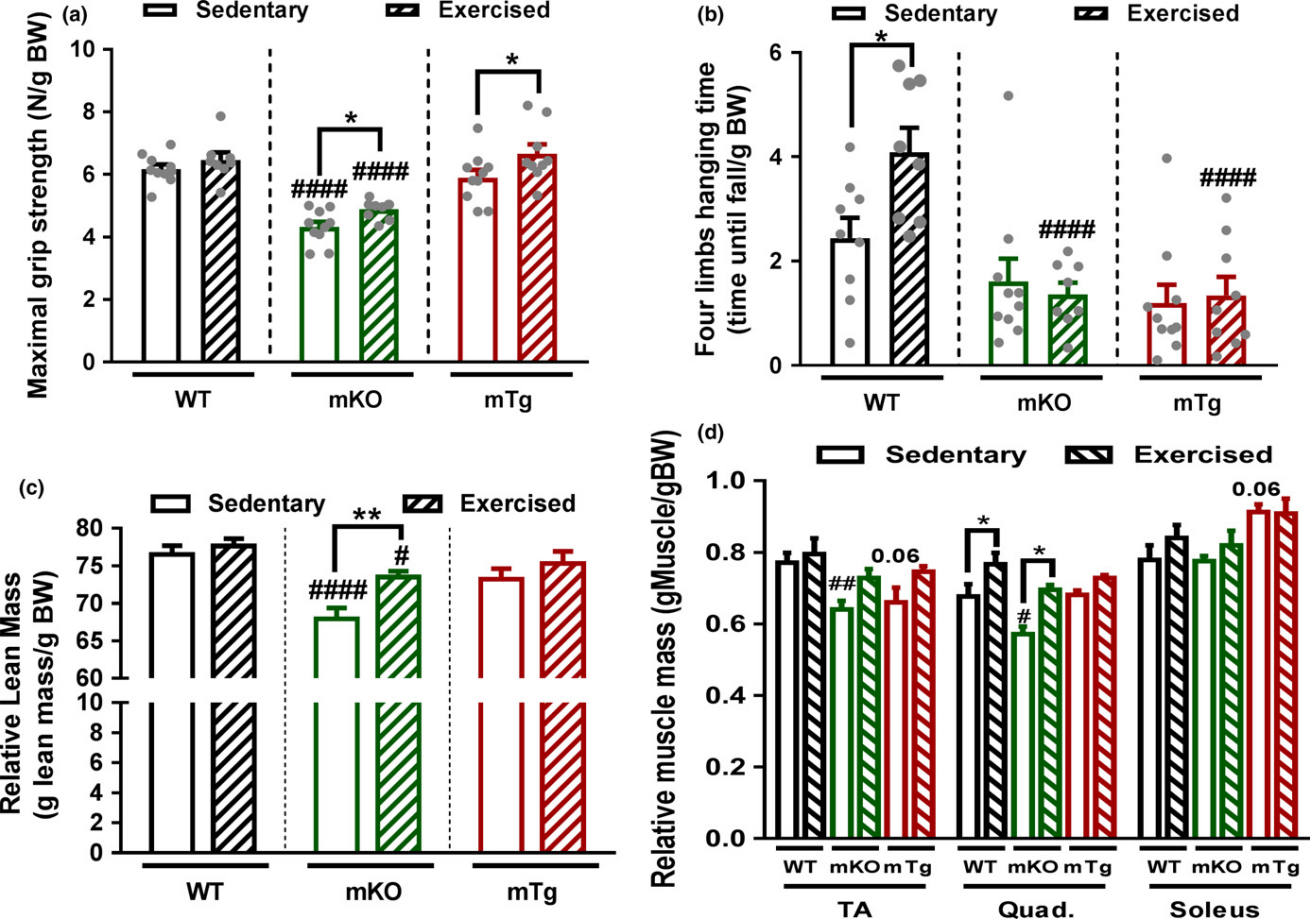
Exercise reduces premature sarcopenia in the absence of PGC‐1α (a) Maximal gip strength (*n* = 8–10). (b) Four limbs hanging time (*n* = 8–10). (c) Lean relative to body mass (*n* = 8–10). (d) muscle mass relative to body weight (*n* = 5–6). Values are mean ± *SEM*. *p < .05; **p < .01; indicate statistically significant differences between sedentary and exercised animals of the same genotype, ^#^ *p* < .01; ^##^ *p* < .01; ^####^ *p* < .001 indicate statistically significant differences between genotypes for sedentary and exercised animals

Interestingly, PGC‐1α overexpression blunts the age‐related decrease in endurance capacity similarly to exercise, while deletion of the transcription factor did not affect this drop. Besides reduced muscle function, a loss of muscle force and mass are the key hallmarks of sarcopenia. An assessment of grip strength accordingly revealed an age‐dependent diminution in maximal grip strength and four limbs hanging time in all genotypes (Figure 3a,b), correlating with loss of relative mass of individual muscles (Figure 3d). The differential increase in body weight in the different genotypes (Fig. S2a) suggests that those changes might be at least in part driven by an increased body mass. However, the absence of changes in absolute muscle mass, overall lean mass or fiber size in WT animals (Fig. S2b and Figure 3c–e), suggests that these mice are only at the stage of very early onset of muscle aging. Strikingly, old mice deficient for muscle PGC‐1α presented an accelerated age‐related reduction in four limbs hanging time, in addition to diminished maximal grip strength, lean mass, muscle weights, and fiber size compared to WT animals, highlighting the development of premature signs of muscle aging in those mice that might also be in part mediated by an increased body weight (Fig. S2a). Although mTg animals exhibited a slight age‐related loss of lean mass, it was not significantly different from WT animals and the impact of PGC‐1α overexpression on individual muscle weight reduction was muscle bed‐specific. Even though endurance exercise training is not the most efficient intervention to improve muscle mass, exercise partially rescued the loss of maximal grip strength, lean mass, and muscle weight in old mKO mice, although these parameters remained lower compared to those in the trained and untrained WT group (Figure 4a,b,d). Interestingly, exercise improved four limbs hanging time in old WT mice, but not in mice lacking muscle PGC‐1α, possibly because of their reduced maximal grip strength and muscle mass (Figure 4b). Surprisingly, this absence of an exercise effect on mass was also found in mice overexpressing PGC‐1α. Nevertheless, PGC‐1α overexpression potentiated the effect of exercise on maximal grip strength in old animals (Figure 4a).

Muscle mass and function are important predictors of metabolic homeostasis. Accordingly, the age‐associated decrease in muscle mass and function was linked to a deterioration of glucose tolerance in WT and mTg animals, whereas young mKO mice already exhibited a glucose tolerance similar to that of 2‐year‐old animals (Fig. S3a). The higher baseline blood glucose levels of young compared to old mTg animals are the main drivers of the age‐related increased AUC in this genotype. Further analysis showed that down‐ and up‐regulation of PGC‐1α both lowered diastolic blood pressure. However, more importantly, blood pressure increased in old WT and mKO, but not in mTg mice (Fig. S3b). Exercise elicited a trend toward reversing age‐associated glucose intolerance in the WT group and failed to significantly improve glucose clearance in mTg animals, whereas it did not lead to any changes in mKO mice (Fig. S4a). Diastolic blood pressure was not influenced by exercise in any of the genotypes (Fig. S4b).

### 2.4 Exercise and PGC‐1α improve age‐related impairments in motor coordination and balance

**Figure 5:**
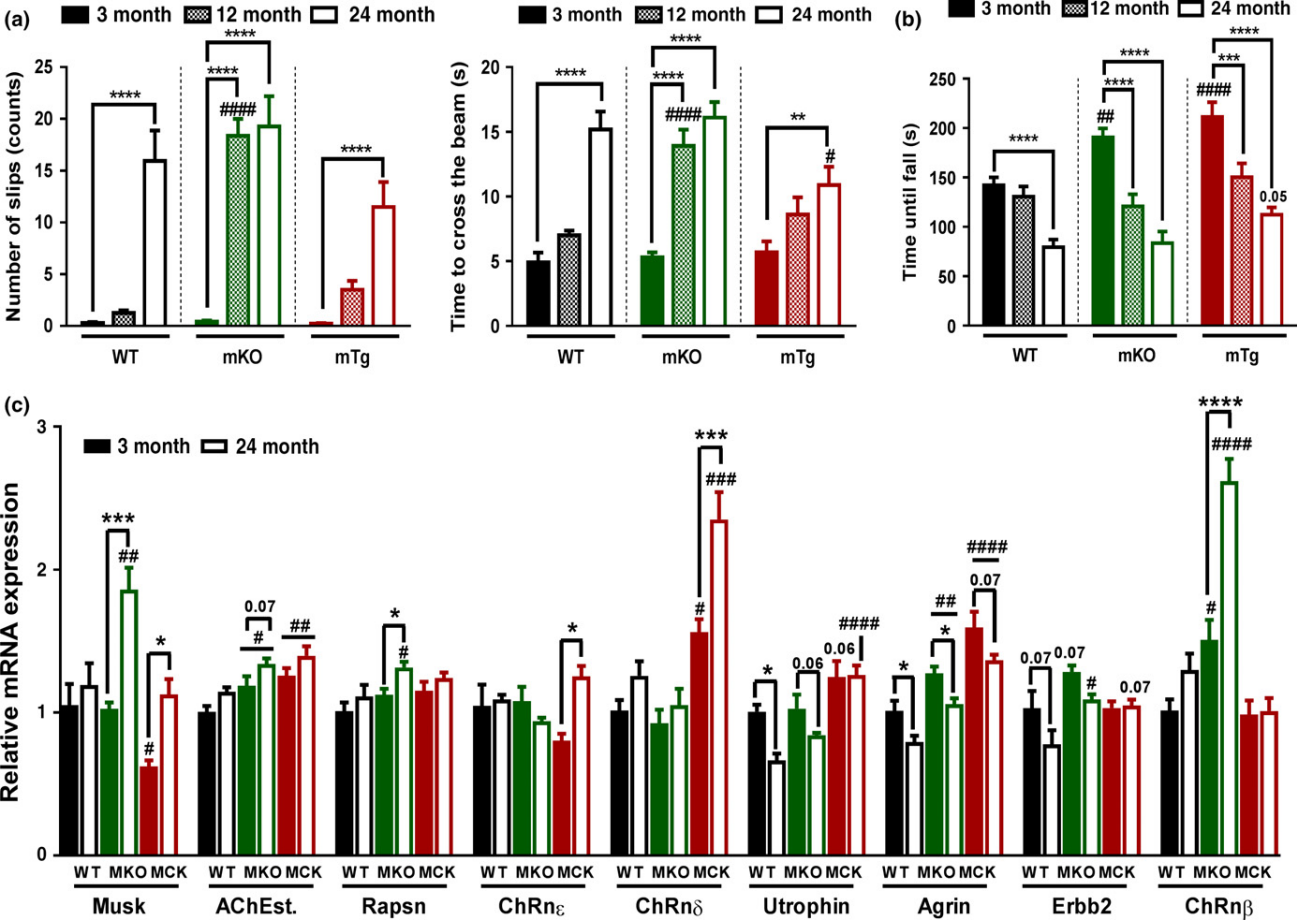
PGC‐1α delays locomotor dysfunction during aging (a) Balance performances measured during balance beam crossing (*n* = 10–12). (b) Motor coordination of mice challenged with a Rotarod (*n* = 10–12). (c) Relative mRNA levels of neuromuscular junction genes measured in tibialis anterior muscles (*n* = 5–6). Values are mean ± *SEM*. *p < .05; **p < .01; ***p < .001; ****p < .0001 indicate statistically significant differences between young and old animals of the same genotype. ^#^ *p* < .01; ^##^ *p* < .01; ^###^ *p* < .01; ^####^ *p* < .001 indicate statistically significant differences between genotypes of age‐matched animals

**Figure 6:**
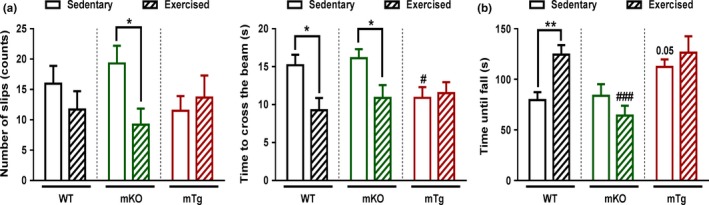
PGC‐1α elevation mimic exercise to improve locomotor function in aged mice (a) Balance performances measured during balance beam crossing (*n* = 8–10). (b) Motor coordination of mice challenged with a Rotarod (*n* = 10). Values are mean ± *SEM*. *p < .05; **p < .01; indicate statistically significant differences between sedentary and exercised animals of the same genotype. ^#^ *p* < .01; ^###^ *p* < .01; indicate statistically significant differences between genotypes for sedentary and exercised animals

The detrimental effects of sarcopenia in elderly individuals are compounded by a loss of motor coordination and balance. We thus recorded how motor coordination and balance in old mice can be modulated by exercise and muscle PGC‐1α using balance beam‐ and Rotarod‐based tests. The number of foot slips and the time needed to cross the balance beam rise dramatically with increasing age in all three genotypes (Figure 5a). Strikingly however, the mKO mice exhibited the phenotype of 24‐month‐old WT mice already at the age of 12 months. Inversely, the age‐dependent reduction in both motor coordination and balance performance was blunted in mTg animals. Curiously, young mKO and mTg mice performed better in Rotarod tests at young age (Figure 5b). Again, a more precipitous drop in performance was observed in mKO animals at 12 months of age. Further analysis identified disparate age‐related modulations of neuromuscular junction transcripts across the different genotypes (Figure 5c), suggesting an alternative reshaping of the neuromuscular junction according to PGC‐1α expression levels. Endurance exercise training of old animals inversely improved the number of foot slips and the time to cross the balance beam in mKO mice (Figure 6a). Moreover, the time to cross the balance beam was also reduced in trained WT animals to the level observed in sedentary mTg mice. Similarly, Rotarod performance in WT mice after exercise reached the level of sedentary mTg animals (Figure 6b). Strikingly, mKO mice did not respond to exercise in the Rotarod‐based assessment of motor coordination.

## 3 DISCUSSION

Exercise is widely accepted as one of the most efficient treatments against the effects of systemic and muscle‐specific aging (Garatachea et al., 2015; Gremeaux et al., 2012). We show here that even when performed only at old age, exercise is sufficient to prevent or even improve many age‐related deteriorations. While evidence has been presented that demonstrate a role for PGC‐1α modulation in the muscle metabolism and inflammation of old muscle‐specific knockout animals (Sczelecki et al., 2014), we now have studied the role of this coactivator in muscle aging in the context of exercise as well as both gain of function and loss of function. Most surprisingly, we observed a strong involvement of muscle PGC‐1α in the control of motor coordination and balance. An improvement of these parameters has been shown in exercise, which exerts pleiotropic effects on different organs and cell types. Our findings now demonstrate that muscle‐intrinsic changes triggered by PGC‐1α are sufficient to affect motor coordination and balance in old mice. Prior work revealed that central nervous system‐specific expression of PGC‐1α is essential for motor function (Lucas et al., 2012; Zhao et al., 2011). Our present data would suggest that skeletal muscle possibly initiates a retrograde signaling to the nervous system with a similar outcome, but further studies would be needed to test this hypothesis. Analogous findings have been reported in the remodeling of the neuromuscular junction (Arnold et al., 2014). Several muscle‐intrinsic and muscle‐extrinsic factors contribute to maintenance of motor coordination and balance besides neuronal changes, including motor planning, strength, and endurance, all of which are decreased with age (Garatachea et al., 2015). Thus, reduced muscle mass in aged mKO‐PGC‐1α mice compared to age‐matched WT animals and the ensuing reduction in maximal grip strength could be important for balance and motor coordination (Schultz, Ashton‐Miller, & Alexander, 1997). Additionally, muscle fiber contraction speed is essential to counteract disequilibrium or to initiate adaptations to changes in motor planning (Schultz et al., 1997). This parameter is highly dependent on calcium homeostasis (Berchtold, Brinkmeier, & Muntener, 2000). Interestingly, muscle PGC‐1α remodels sarcoplasmic reticulum calcium handling (Summermatter et al., 2012) and thereby possibly affects muscle contraction and ultimately locomotor function. Furthermore, neuromuscular junctions, which constitute the interface allowing a proper communication between motor neurons and muscles, are severely deteriorated during aging (Tintignac, Brenner, & Ruegg, 2015). This impairment causes inappropriate or inefficient muscle contraction, as well as the defect of retrograde signaling from the muscle to the nerves, thereby dramatically impacting muscle force, motor coordination, and balance. Interestingly, muscle PGC‐1α overexpression ameliorates neuromuscular junction morphology and function, which might also contribute to the improvement of age‐related motor dysfunction (Arnold et al., 2014). Likewise, the modulation of expression of several neuromuscular junction genes found in this study implies an age‐ and genotype‐dependent remodeling of this structure that could affect the motor function of our animals during aging. However, a more careful and comprehensive analysis of the morphology and function of the neuromuscular junction will have to be performed to reach conclusions about a potential protective effect of PGC‐1α against neuromuscular junction degeneration in old muscle. Of note, ablation of muscle PGC‐1α abolished the effect of exercise on motor coordination and bunted the exercise‐associated increase in endurance. Conversely, overexpression of muscle PGC‐1α boosted the effect of exercise on both muscle endurance and maximal grip strength. Those data indicate that the exercise effects on motor coordination, endurance, and maximal grip strength, at least as assessed in our tests, are modulated upon alteration of muscle PGC‐1α levels. Interestingly however, the beneficial effect of PGC‐1α on age‐related and exercise‐mediated muscle adaptations is limited to specific alteration of muscle functions and thus is insufficient to control the entire biological program. For example, while PGC‐1α deletion abrogated the exercise‐related increase in Rotarod performance, improvements in balance induced by exercise were unaffected in these mice. Similarly, opposite to four limbs hanging time, the boosts in muscle mass and maximal grip strength postexercise are not prevented by the absence of PGC‐1α indicating that other elements can compensate for the genetic ablation of this gene. Finally, while overexpression of PGC‐1α strikingly ameliorated motor and muscle functions, no change in maximal grip strength and mass was observed. These data show that PGC‐1α plays a complex and nuanced role in muscle aging as genetic ablation and transgenic overexpression affect very specific aspects of muscle biology in old mice.

In our experimental paradigm, exercise did not affect whole‐body muscle mass and only quadriceps muscle mass in our WT animals. In line with these results, maximal grip strength was likewise unaffected. Resistance exercise would probably be more effective in improving these parameters. Nevertheless, endurance exercise training increased four limbs hanging time and running capacity in WT mice. Endurance exercise might thus be sufficient to improve sarcopenia at a later age and upon higher muscle strength loss. This is supported by the partial restoration of both muscle mass and maximal grip strength in mKO mice, which displayed an advanced stage in sarcopenia.

Interestingly, even though exercise improved PGC‐1α muscle gene expression in some muscles, no changes in mRNA levels of mitochondrial genes were observed. In contrast, an elevation in OXPHOS protein levels correlated with the increased endurance performance of these mice, similar to what has been described in young compared to old humans (Robinson et al., 2017). Strikingly, PGC‐1α muscle deletion blunted exercise induction of OXPHOS proteins and PGC‐1α overexpression potentiated the effects of exercise on mitochondrial mass and on the proportion of oxidative fibers. Considering the impact of age on both type I and II muscle fibers (Purves‐Smith, Sgarioto, & Hepple, 2014) and the proportion of 50% of type I fibers in human limb muscle, an analysis of the role of PGC‐1α in the protection of type I fiber in mouse soleus muscle will be of interest. Nevertheless, our data demonstrate that exercise improves mitochondrial fitness and oxidative metabolism in a PGC‐1α‐dependent manner, also in old muscles. Correlating with our results, previous work showed that global and muscle‐specific PGC‐1α deletion prevents exercise‐associated amelioration of mitochondria (Leick, Lyngby, Wojtaszewski, & Pilegaard, 2010). Of note, the mitochondrial number in WT and mKO animals, at least as suggested by the quantification of mitochondrial DNA, did not reflect the close link between mitochondria and endurance capacity (Figure 1d). This suggests that increased OXPHOS protein levels per individual mitochondria could contribute to the improved exercise performance.

Skeletal muscle is the largest tissue responding to insulin and therefore is a primary site for whole‐body glucose uptake, utilization, and storage. Glucose intolerance and increased blood pressure represent hallmarks of the age‐related metabolic syndrome (Hildrum, Mykletun, Hole, Midthjell, & Dahl, 2007). In line with the aggravated glucose intolerance in old mKO animals described previously (Sczelecki et al., 2014), we observed that absence of muscle PGC‐1α accelerated glucose intolerance during aging. Even though no overt amelioration in glucose tolerance was observed in the mTG mice, overexpression of muscle PGC‐1α abolished the age‐associated increase in blood pressure. Therefore, collectively, muscle PGC‐1α reduces the development of an age‐related metabolic syndrome. As the metabolic syndrome is highly predictive for heart disorders and failure (Perrone‐Filardi et al., 2015), it would be interesting to study the effect of exercise combined with modulation of muscle PGC‐1α on the aging heart.

In conclusion, our findings demonstrate that many of the beneficial effects of exercise involve the action of muscle PGC‐1α. Of note, some parameters show an additive effect of endurance exercise with overexpression of PGC‐1α, suggesting an obvious therapeutic relevance. While an improvement of motor coordination and balance could have been expected from the exercise intervention, the striking effect of overexpression and ablation of muscle PGC‐1α reveal a hitherto unsuspected contribution of muscle fibers to these neuronally controlled processes. More work is required to identify the molecular mechanisms that underlie this observation. Nevertheless, our data highlight the importance of proper muscle function on aging‐related deteriorations that transcend the immediate effects on muscle fibers.

## 4 MATERIAL AND METHODS

### 4.1 Animals

Mice with muscle ablation (mKO) and overexpression (mTg) of PGC‐1α were described previously (Handschin, Choi et al., 2007; Lin et al., 2002). C57/Bl6 wild‐type (WT) mice were obtained from Janvier (Janvier sas, cs 4105, le genest St‐isle f‐53941, St Berthevin Cedex). Animals were fed ad libitum with regular chow diet (Provimi 3432) and kept under a 12‐h/12‐h light–dark cycle (06:00 to 18:00) at 23°C. Studies were performed with 3‐, 12‐, and 24‐month‐old male mice (3, 12, and 24 months). At 21 months, groups of mice were trained on a treadmill during 12 weeks, 3 times per week, for 30 min. Maximal speed was determined prior to the beginning of the endurance exercise training by an exhaustion test. The exercise protocol started at 50% of maximal speed in the first week and was gradually increased each week to reach 80%. All experiments were performed in accordance with the federal guidelines for animal experimentation and were approved by the Kantonales Veterinäramt of the Kanton Basel‐Stadt.

### 4.2 Mice phenotyping

For all behavioral experiments, mice were acclimatized to the room for 1 hr prior to the tests and experimenters were blinded for the animal genotype and the treatment wherever possible.

#### 4.2.1 Balance performance

Mice were placed on one side of an inclined 6‐mm‐diameter beam and were motivated to walk across the bar toward a red house used as a positive stimulus. A lamp was placed at the starting point as an adverse stimulus. Mice were acclimatized to the apparatus for one day with 3 beam crossing including 15 s of rest in the red house between trials. Time required to cross the beam and number of foot slips made during the crossing were recorded during the 3 following days with 3 trials per day.

#### 4.2.2 Motor coordination and planning

Mice were placed on a horizontally oriented, rotating cylinder (rod). For 3 consecutive days, mice were acclimatized 3 × 1 min per day on the Rotarod instrument set to rotate at 5 rpm. On the 5 following days, mice were tested with accelerating speed (5–68 rpm in 7 min with directional reversal). Mice were tested three times in a row with 10 min of rest between each trial. The time the mice spend on the rod before falling was recorded and averaged over the 5 days.

#### 4.2.3 Maximal grip strength and four limbs hanging time

Maximal grip strength was recorded using a grip strength meter (Chatillon). Five measurements were performed with 1‐min recovery periods between the repetitions. The maximum value obtained was used for the analysis. Four limbs hanging time was measured by placing mice on an elevated inverted grid and measuring the maximum time until the mice released and fell.

#### 4.2.4 Endurance exercise capacity

Mice were acclimatized to treadmill running (Columbus Instruments) for 2 days. Acclimatization was performed with an inclination of 5% at a speed of 8 m/min for 5 min followed by 5 min of 10 m/min. The exhaustion test was performed one day after acclimatization, starting at a speed of 4.8 m/min and a subsequent an increased by 1.6 m/min every 3 min until a speed of 29 m/min was reached. When mice reached exhaustion, the maximal running distance was recorded. Blood was drawn from the tail vein before and 10 min after the test, and blood lactate levels were measured with a lactate plus meter (Nova biomedical).

### 4.3 Metabolism measurements

#### 4.3.1 Body composition

Body composition was assessed with an EchoMRI‐100 analyzer (EchoMRI Medical Systems). Fat and lean mass were adjusted to body weight.

#### 4.3.2 Glucose homeostasis

All mice were acclimatized to handling before the experiment. Mice were fasted for 16‐hr overnight before intraperitoneal injection of a bolus of 2 g (glucose)/kg (body weight). Blood glucose levels were recorded from the tail vein with a glucose meter (Accu‐Chek, Roche Diagnostics) at 0, 15, 30, 45, 60, 90, and 120 min after glucose injection. The AUC was calculated as described (Heikkinen, Argmann, Champy, & Auwerx, 2007).

#### 4.3.3 Blood pressure

Blood pressure was measured with a tail cuff BP‐2000 Blood Pressure Analysis System (Visitech Systems) on 5 consecutive days. Five premeasurements and 15 measurements were performed in a row. The average values of the 15 measurements over the 5 days were used for analysis.

### 4.4 Mouse muscle preparation

Body weight of the mice was measured before sacrifice by CO2 inhalation. Muscles were directly collected and weighed before being either snap‐frozen in liquid nitrogen for protein and RNA extraction, embedded in 7% tragacanth using cooled‐isopentane for cryosection staining.

### 4.5 Mitochondria DNA copy number and gene expression

#### 4.5.1 Genomic DNA

Powdered muscles were incubated overnight at 55° in lysis buffer (10 mm Tris‐HCl, 1 mm EDTA, 0.1% SDS 5% Proteinase). After a 15‐min centrifugation step at 8,000 *g*, residual RNA of the re‐suspended lysate was removed with a RNase A treatment (20 mg/ml) during 30 min at 37°C. RNase A was heat‐inactivated at 95°C for 30 min. Samples were mixed with one volume of phenol/chloroform/isoamyl alcohol (25:24:1) and centrifuged at 8,000 g for 15 min. The aqueous phase was transferred to a fresh tube, and one volume of chloroform was added. After an identical centrifugation step, one volume of isopropanol containing 0.3m of sodium acetate was added. Samples were then kept at −20°C for 30 min. A centrifugation step was repeated at 4°C. DNA pellets were washed 2 times with 1 ml of ice‐cold 75% ethanol and finally recovered in ddH2O. 0.1 μg of gDNA was analyzed by qPCR to measure mitochondrial DNA copy numbers.

#### 4.5.2 RNA extraction and qPCR

Lysing matrix tubes (MP Biomedicals 6913‐500) and TRI Reagent (Sigma‐Aldrich T9424) were used to isolate RNA from crushed muscle. NanoDrop 1000 spectrophotometer (Thermo Scientific) was used to measure RNA concentration and purity to ensure that the ratios of 260/280 and 260/230 were above 1.8. An Agilent Bioanalyzer (Agilent 2100 Bioanalyzer, Agilent Technologies) determined the level of RNA degradation. DNA was removed with RNase‐free DNase (Invitrogen 18068‐015), and one microgram of RNA was used for reverse transcription using SuperScript II reverse transcriptase (Invitrogen 18064‐014).

Relative DNA copy number or mRNA levels were determined using a Light Cycler 480 system (Roche Diagnostics) with FastStart Essential DNA probe master mix (Roche Diagnostics 06402682001). Relative mRNA quantification was performed with the ΔΔCT method using the TATA binding protein (TBP) gene as reference. Mitochondrial DNA copy number was evaluated by normalizing the average of COX1 ATP6 and ND1DNA copy number with the average copy number of the nuclear genes beta globin and 34B6. Beta globin, 34B6, and TBP levels were similar in all samples. Primer used is listed in Table S1.

### 4.6 Immunostaining

Eight‐μm muscle cryosections were cut with a cryostat (Leica, CM1950). Sections were blocked for 30 min at ambient temperature in PBS containing 0.4% Triton X‐100 and 10% goat serum. Fiber types and cell membranes were labeled with primary and secondary antibodies for one hour at room temperature in PBS with 10% goat serum (antibody dilutions and information are described in Table S2). Incubation with secondary antibodies was performed in the dark. All secondary antibodies were from Life technologies. Three washes of 5 min in PBS were performed before and after antibody incubation. Nuclei were stained using ProLong Gold Antifade mounting medium with Dapi (Life Technologies, P36931). Images were taken using the FEI MORE microscope with a 20× magnification lens keeping the same acquisition settings for all compared samples. Fiber type percentages and diameters were quantified in whole muscle sections using the ImageJ and cell^P software (Olympus Soft Imaging Solution) with unaltered images. Representative images were, however, adjusted for brightness and contrast for visualization purposes.

### 4.7 Protein levels

Total protein was recovered from quadriceps muscles as described in Perez‐Schindler, Summermatter, Santos, Zorzato, and Handschin (2013). Equal amounts of protein were migrated on mini‐TGX 4‐20% stain free precast gel (Bio‐Rad, 4568096) and labeled with the in‐gel trihalo compounds under UV for 1 min. Gel and nitrocellulose membranes were allowed to equilibrate for 5 min in transfer buffer. Protein transfer was performed at a constant voltage of 100 V for 1 hr. Membranes were blocked for 1 hr at room temperature with 5% BSA diluted in TBS‐T and washed 2× for 5 min with TBS‐T. Membranes were then incubated overnight at 4°C with primary antibodies (Abcam, ab110413, 1/1000) diluted in TBS‐T containing 0.02% sodium azide and 3% BSA. Membranes were washed 3× for 5 min with TBS‐T and incubated 1 hr at room temperature with peroxidase‐conjugated secondary antibodies (Dako, P0260, 1/10000) diluted in TBS‐T plus 3% BSA. The same washing step was repeated before antibody binding was revealed using an enhanced chemiluminescence HRP substrate detection kit (Thermo Scientific, 34076) and imaged using a Fusion FX imager. Total protein content was determined using the trihalo compounds labeling. Two reference samples were loaded in the different gels for intergel normalization. Proteins’ quantification was performed using the Fusion FX software.

### 4.8 Statistical analysis

Data were analyzed with two‐way ANOVA (GraphPad Prism software). Sidak posttests were used to do multiple comparison analysis following two‐way ANOVA. All data are plotted as mean ± *SEM*.

## AUTHOR'S CONTRIBUTION

J.F.G. and C.H. designed the study, interpreted the data, and wrote the manuscript; J.F.G., G.S., and S.S. performed the experiments. All authors approved the final version of the manuscript.

## CONFLICT OF INTEREST

The authors declare no conflict of interest.
