# Supplementary material for: PGC‐1α affects aging‐related changes in muscle and motor function by modulating specific exercise‐mediated changes in old mice
Source: Aging Cell. 2017 Oct 25;17(1):e12697. doi: 10.1111/acel.12697 (PMC5770876; doi:10.1111/acel.12697)
Supplement: Supplementary file 1 [file ACEL-17-na-s001.pdf]

<u>Supplemental Tables and Figures</u>

Table S1: qPCR primer sequences

Gene name	forward sequence	reverse sequence
**βglobin**	GAAGCGATTCTAGGGAGCAG	GGAGCAGCGATTCTGAGTAGA
**36B4**	ACTGGTCTAGGACCCGAGAAG	TCAATGGTGCCTCTGGAGATT
**ATP6**	AGTATGAGCTGGAGCCGTAATTACA	TGGAAGGAAGTGGGCAAGTG
**COX1**	TGCTAGCCGCAGGCATTACT	GCGGGATCAAAGAAAGTTGTG
**COX5a**	CGCCGCTGTCTGTTCCAT	AAACTCCTCATCTGTCTCGTGTGA
**COX5b**	CTTCAGGCACCAAGGAAGAC	TTCACAGATGCAGCCCACTA
**ERRα**	CGGTGTGGCATCCTGTGA	CTCCCCTGGATGGTCCTCTT
**ND1**	TCTGCCAGCCTGACCCATA	GGGCCCGGTTTGTTTCTG
**NRF1**	TCCCCCGAGGACACTTCTT	ATCAGCTGCCGTGGAGTTG
**PGC-1α**	AGCCGTGACCACTGACAACGAG	GCTGCATGGTTCTGAGTGCTAAG
**SDH**	GCTGGTGTGGATGTCACTAAGG	CCCACCCATGTTGTAATGCA
**TBP**	ATATAATCCCAAGCGATTTGC	GTCCGTGGCTCTCTTATTCTC
**MuSK**	ATCCCAACCCCATGTACCAG	CCTTAAGCATCTTCACGGCC
**AchEst**	CGGAGGCTCTCATCAATACTGG	GGGACCCCGTAAACCAGAAAG
**Rapsyn**	AGACAGCCTTCCCTCGGTA	CTTCAGCTAAGTCCTGGGCT
**Agrn**	ATGGCAAGACACCCTCACTG	TCCTTGCAGTCTCCCCAAAC
**Erbb2**	AAGAGGTCACAGCTGAGGAC	AAAATGCCAGGCTCCCAAAG
**AChRβ**	CATCATCGCTCACCCCAC	ACGGTCCACAACCATGGC
**AChRε**	TGTTTGAGGGTCAGAGGCAT	AGTTCCTCTCCAGTGGCTTC
**AChRα**	AGTCCAATAACGCCGCTGAG	TTTCCTAGCGATGGCTATGG
**AChRδ**	ACAAAGTGGATGTCGCCCTA	TCTCTGGTAGCCACACCATG
**AChRγ**	GACCAACCTCATCTCCCTGA	GAGAGCCACCTCGAAGACAC
**Utrophin**	GAGGCTCGCTTCTACATGCT	TGATGACACCATCGCCAGAC

Table S2: Antibody information

Primary antibodies				Secondary antibodies		
Product#	Company	Labelling	Dilution	Product#	Labelling	Dilution
BA-F8	DSHB	m IgG b2 MHC type 1	1:25	A-21242	AF647 IgG GaM	1:250
SC-71	DSHB	m IgG1 MHC type 2a	1:200	A-21124	AF568 IgG1 GaM	1:100
BF-F3	DSHB	m IgM MHC type 2b	1:100	A-21042	AF488 IgM GaM	1:400
L9393	Sigma	r IgM Laminin	1:5000	A-11008	AF488 IgG GaR	1:500

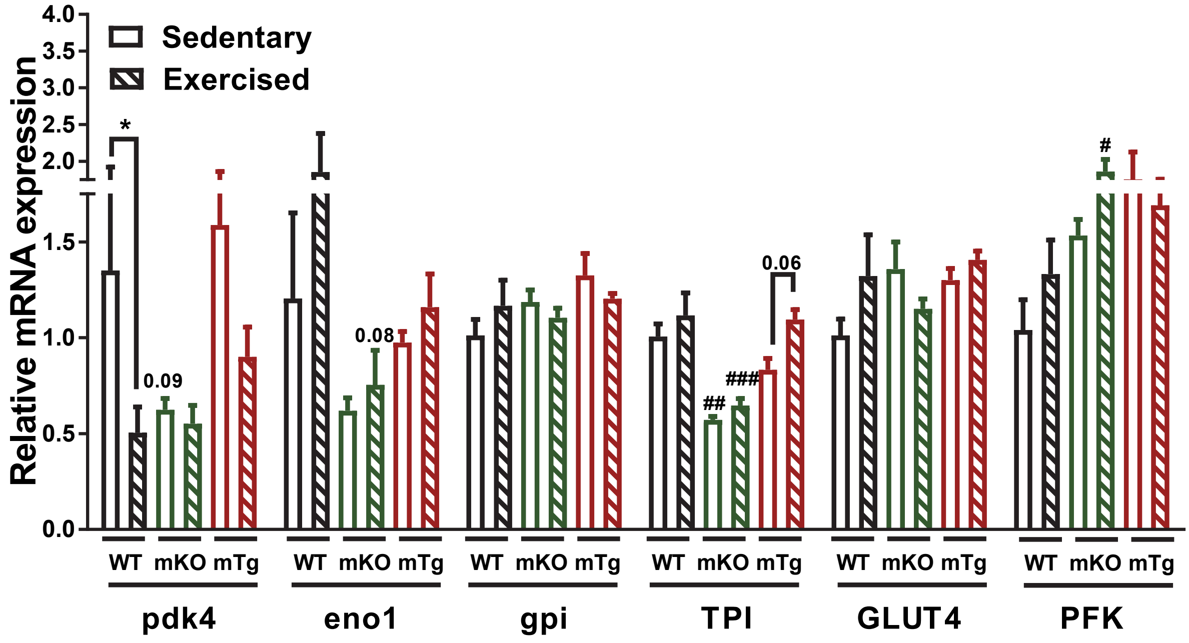

Fig. S1 : Exercise-dependent regulation of glycolysis genes. Relative quadriceps mRNA levels of genes involved in lactate regulation and glycolysis (n=4-6). Values are mean ± SEM. *P < 0.05; **P < 0.01; ***; P < 0.001; ****p<0.0001 indicate statistically significant differences between young and old animals of the same genotype, # p<0.01; ## p<0.01; ### p<0.01; #### p<0.001 indicate statistically significant differences between genotypes of age-matched animals.

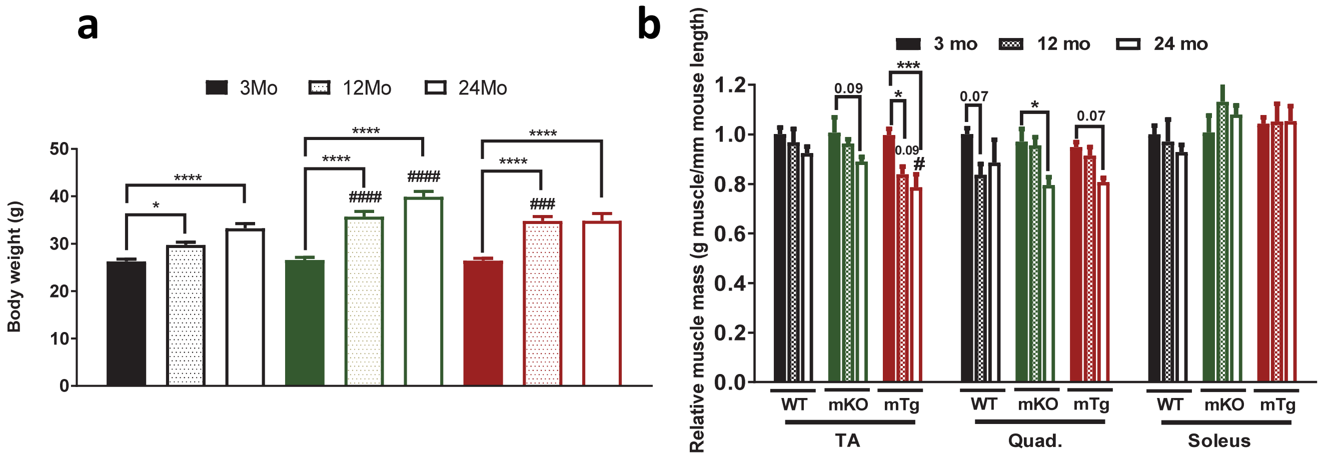

Fig. S2 : Body weight (n=10-12) (a) and absolute mass of isolated muscles (n=5-6) (b) Values are mean ± SEM. *P < 0.05; **P < 0.01; ***; P < 0.001; ****p<0.0001 indicate statistically significant differences between young and old animals of the same genotype, # p<0.01; ## p<0.01; ### p<0.01; #### p<0.001 indicate statistically significant differences between genotypes of age-matched animals.

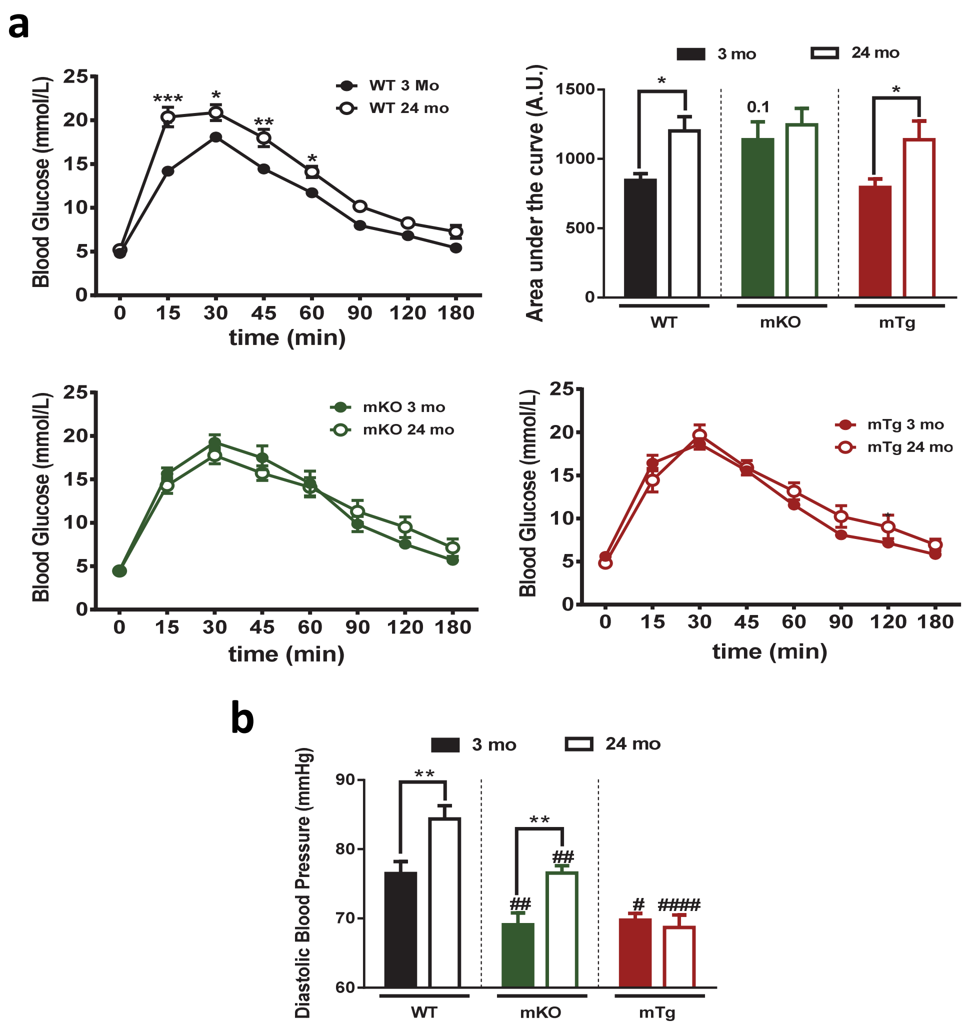

Fig. S3 : PGC-1α improves systemic aging (a) Glucose tolerance test curves and area under the curves (n=10) **(b)** Diastolic blood pressure (n=10). Values are mean ± SEM. *P < 0.05; **P < 0.01; ***; P < 0.001; ****p<0.0001 indicate statistically significant differences between young and old animals of the same genotype, # p<0.01; ## p<0.01; ### p<0.01; #### p<0.001 indicate statistically significant differences between genotypes of age-matched animals.

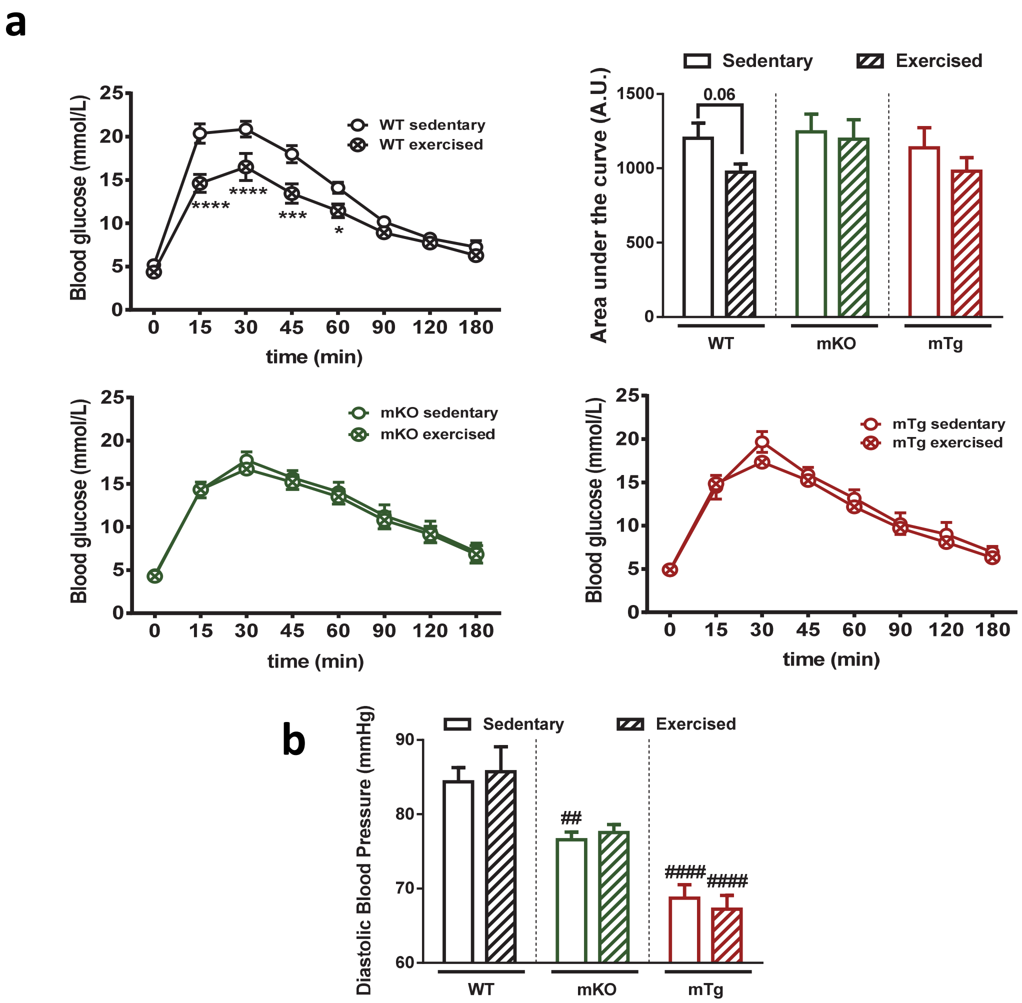

Fig. S4 : Exercise and PGC-1α modulation impacts on systemic aging (a) Glucose tolerance test curves and area under the curves (n=8-10) **(b)** Diastolic blood pressure (n=10-12) Values are mean ± SEM. *P < 0.05; **P < 0.01; ***; P < 0.001; ****p<0.0001 indicate statistically significant differences between sedentary and exercised animals of the same genotype, # p<0.01; ## p<0.01; ### p<0.01; #### p<0.001 indicate statistically significant differences between genotypes for sedentary and exercised animals.
